# Efficacy of Statin Therapy in Pulmonary Arterial Hypertension: A Systematic Review and Meta-Analysis

**DOI:** 10.1038/srep30060

**Published:** 2016-07-22

**Authors:** Magdalena Rysz-Górzynska, Anna Gluba-Brzózka, Amirhossein Sahebkar, Maria-Corina Serban, Dimitri P. Mikhailidis, Sorin Ursoniu, Peter P. Toth, Vera Bittner, Gerald F. Watts, Gregory Y. H. Lip, Jacek Rysz, Alberico L. Catapano, Maciej Banach

**Affiliations:** 1 Department of Hypertension, Chair of Nephrology and Hypertension, Medical University of Lodz, Poland; 2 Healthy Aging Research Centre (HARC), Medical University of Lodz, Lodz, Poland; 3 Department of Nephrology, Hypertension and Family Medicine, Chair of Nephrology and Hypertension, Medical University of Lodz, Poland; 4 Biotechnology Research Center, Mashhad University of Medical Sciences, Mashhad, Iran; 5 Metabolic Research Centre, Royal Perth Hospital, School of Medicine and Pharmacology, University of Western Australia, Perth, Australia; 6 Department of Epidemiology, University of Alabama at Birmingham, Birmingham, AL, USA; 7 Department of Functional Sciences, Discipline of Pathophysiology, “Victor Babes” University of Medicine and Pharmacy, Timisoara, Romania; 8 Department of Clinical Biochemistry, Royal Free Campus, University College London Medical School, University College London (UCL), London, UK; 9 Department of Functional Sciences, Discipline of Public Health, “Victor Babes” University of Medicine and Pharmacy, Timisoara, Romania; 10 Preventive Cardiology, CGH Medical Center, Sterling, Illinois, USA; 11 The Johns Hopkins Ciccarone Center for the Prevention of Heart Disease, Baltimore, MD, USA; 12 Lipid Disorders Clinic, Cardiovascular Medicine, Royal Perth Hospital, School of Medicine and Pharmacology, University of Western Australia, Perth, WA, Australia; 13 University of Birmingham Institute of Cardiovascular Sciences, City Hospital, Birmingham, UK; 14 Department of Pharmacological and Biomolecular Sciences University of Milan and IRCCS Multimedica Milano Italy

## Abstract

Since the evidence regarding statin therapy in PAH has not been conclusive, we assessed the impact of statin therapy in PAH through a systematic review and meta-analysis of available studies. We searched selected databases up to August 1, 2015 to identify the studies investigating the effect of statin administration on PAH. Meta-analysis was performed using either a fixed-effects or random-effect model according to *I*^*2*^ statistic. Meta-analysis of 8 studies with 665 patients did not suggest any significant improvement in 6-min walking distance (6MWD) by statin therapy (weighed mean difference [WMD]: −6.08 m, 95% confidence interval [CI]: −25.66, 13.50, *p* = 0.543; Q = 8.41, I^2^ = 28.64%). Likewise, none of the other indices including pulmonary arterial pressure (WMD: −0.97 mmHg, 95%CI: −4.39, 2.44, *p* = 0.577; Q = 14.64, I^2^ = 79.51%), right atrial pressure (WMD: 1.01 mmHg, 95%CI: −0.93, 2.96, *p* = 0.307; Q = 44.88, I^2^ = 95.54%), cardiac index (WMD: 0.05 L/min/m^2^, 95%CI: −0.05, 0.15, *p* = 0.323; Q = 3.82, I^2^ = 21.42%), and pulmonary vascular resistance (WMD: −1.42 dyn*s/cm^5^, 95%CI: −72.11, 69.27, *p* = 0.969; Q = 0.69, I^2^ = 0%) was significantly altered by statin therapy. In conclusion, the results of the meta-analysis did not show a statistically significant effect of statin therapy in the improvement of 6MWD, pulmonary arterial pressure, right atrial pressure, cardiac index and pulmonary vascular resistance.

Pulmonary hypertension (PAH) is defined according to the new 2015 Guidelines of the European Society of Cardiology (ESC) and European Respiratory Society (ERS) as an increase of the mean pulmonary arterial pressure (PAPm) above 25 mmHg at rest, assessed by right heart catheterization (RHC) and pulmonary vascular resistance (PVR) above 3 Wood units^1^. Compared with the 2008 PAH guidelines, the new guidelines eliminated the exercise criterion, added hemodynamic parameters, and defined post-capillary PAH subgroups^1^. The new guidelines categorized various PAH conditions into five groups: pulmonary arterial hypertension, pulmonary hypertension due to left heart disease, pulmonary hypertension due to lung diseases and/or hypoxia, chronic thromboembolic pulmonary hypertension and other pulmonary artery obstructions, and pulmonary hypertension with unclear and/or multifactorial mechanisms^1^. Several prognostic factors such as right ventricular failure, higher World Health Organization functional classification (WHO FC), shorter 6-min walk distance (6MWD) and hemodynamic factors such as right atrial pressure, and brain natriuretic peptide (BNP) levels are useful for PAH prognosis^2^,^3^. Furthermore, the American College of Chest Physicians’ (CHEST) Guideline suggests that the diagnosis of PAH should be made in a systematic and consistent manner with the use of a combination of WHO FC, exercise capacity, echocardiographic, laboratory and hemodynamic variables, and care should be provided by experts in the management of PAH. (Grade CB)^4^. However, the prognosis for patients with PAH remains poor, especially without appropriate treatment^5^.

Current therapy for PAH is based on endothelin receptor antagonists, phosphodiesterase-5 (PDE5) inhibitors, prostacyclin analogues (prostanoids), soluble guanylate cyclase stimulators, and prostacyclin receptor (IP receptor) agonist^6^,^7^. Despite the appearance of novel targets for PAH therapy tested in preclinical and clinical trials such as rituximab, endothelial progenitor cells, specific rho-kinase (ROCK) inhibitors (AT-877ER), FK506 (tacrolimus), fluoxetine, sertraline, paroxetine, bardoxolone methyl, aviptadil or tyrosine kinase inhibitors (nilotinib, sorafenib and imatinib), the diagnosis and treatment of PAH is still difficult^7^.

The pleiotropic effects of statins through induction of endothelial nitric oxide (NO) expression as well as anti-inflammatory and antiproliferative mechanisms possibly confer various clinical advantages beyond the reduction of serum cholesterol levels^8^,^9^. Since statins have been suggested as potential drugs for PAH treatment^10^, we assessed the impact of statin therapy on multiple parameters in PAH in this systematic review and meta-analysis.

## Methods

This study was designed according to the 2009 preferred reporting items for systematic reviews and meta-analysis (PRISMA) guidelines^11^. Due to the study design (meta-analysis) no Institutional Review Board (IRB) approval, as well as no patients’ informed consents were obtained.

### Search Strategy

PubMed-Medline, SCOPUS, Web of Science and Google Scholar databases were searched using the following search terms in titles and abstracts (also in combination with MESH terms): (atorvastatin OR simvastatin OR rosuvastatin OR fluvastatin OR pravastatin OR pitavastatin OR lovastatin OR cerivastatin OR “statin therapy” OR statins OR statin) AND (“pulmonary arterial hypertension” OR “pulmonary hypertension” OR “pulmonary artery hypertension” OR “pulmonary vascular disease” OR “pulmonary heart disease” OR “pulmonary cardiac disease” OR PAH). The wild-card term “*” was used to increase the sensitivity of the search strategy. The literature search was limited to articles published in English. The search was limited to studies in humans. The literature was searched from inception to August 1, 2015. Two reviewers (MRG and AGB) evaluated each article independently. Disagreements were resolved by discussion with a third party (MB).

### Study Selection

Original studies were included if they met the following criteria: (i) being a clinical study with either observational or interventional design, (ii) recruiting patients with a clinical diagnosis of PAH according to echocardiography regardless of the etiology (congenital heart disease, connective tissue disease, chronic thrombembolism or iatrogenic), and, (iii) investigating the impact of statin therapy on a valid disease activity index including 6MWD, pulmonary arterial pressure, right atrial pressure, cardiac index and pulmonary vascular resistance.

Exclusion criteria were: (i) non-clinical studies, (ii) lack of a statin-free control group in the study design, and, (iii) lack of sufficient information on baseline or follow-up indices of PAH.

### Data extraction

Eligible studies were reviewed and the following data were abstracted: 1) first author’s name 2) year of publication 3) study location 4) study design 5) number of participants in the statin and groups 5) age, gender and body mass index (BMI) of study participants 6) type and duration of statin therapy and 7) baseline and follow-up values of PAH-related indices.

Data extraction was performed independently by 2 reviewers; disagreements were resolved by a third reviewer.

### Quality assessment

Methodological quality of the included studies was assessed using the Newcastle-Ottawa Scale (NOS)^12^. In this context, topic items of each eligible study are assessed: that is, the selection of the studied patients, the comparability of the studied populations and the ascertainment of the exposure. A study can be awarded a maximum of one point for each item. Risk-of-bias assessment was performed independently by 2 reviewers; disagreements were resolved by a third reviewer.

### Quantitative Data Synthesis

Meta-analysis was conducted using Comprehensive Meta-Analysis (CMA) V2 software (Biostat, NJ)^13^. Net changes in measurements (change scores) were calculated as follows: measure at end of follow-up − measure at baseline. For single-arm cross-over trials, net change in each efficacy measure was calculated by subtracting the value after control intervention from that reported after treatment. Standard deviations (SDs) of the mean difference were calculated using the following formula: SD = square root [(SD_pre-treatment_)^2^ + (SD_post-treatment_)^2^ − (2R × SD_pre-treatment_ × SD_post-treatment_), assuming a correlation coefficient (R) = 0.5. If the outcome measures were reported in median and range or 95% CI, mean and standard SD values were estimated using the method described by Wan *et al*.^14^. Where standard error of the mean (SEM) was only reported, SD was estimated using the following formula: SD = SEM × sqrt (*n*), where *n* is the number of subjects.

A random-effects model (using DerSimonian-Laird method) and the generic inverse variance method were used to compensate for the heterogeneity of studies in terms of demographic characteristics of populations being studied and also differences in study design and type of statin being studied^15^. Heterogeneity was quantitatively assessed using *I*^*2*^ index. Effect sizes were expressed as weighted mean difference (WMD) and 95% confidence interval (CI). In order to evaluate the influence of each study on the overall effect size, sensitivity analysis was conducted using leave-one-out method, i.e. removing one study each time and repeating the analysis.

### Publication bias

Potential publication bias was explored using visual inspection of Begg’s funnel plot asymmetry, Begg’s rank correlation, and Egger’s weighted regression. Duval and Tweedie “trim and fill” was used to adjust the analysis for the effects of publication bias^16^.

## Results

### Flow and characteristics of included studies

**Figure 1:**
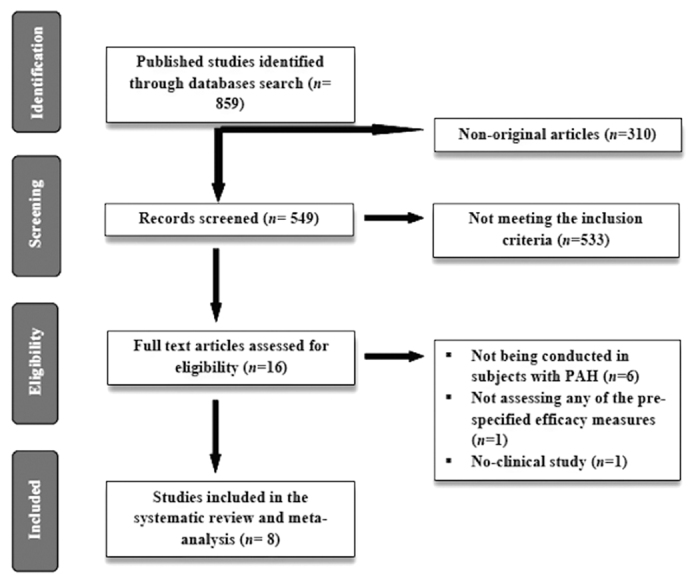
Flow chart of the number of studies identified and included into the meta-analysis.

On the basis of a database search 859 published studies were found, 310 of which were non-clinical or review articles and thus they were not included in further analysis. We screened 549 articles but 533 of them were excluded because they did not meet inclusion criteria. Out of 16 eligible papers, 6 were excluded since they were not conducted in subjects with PAH, one of them did not assess any of the pre-specified efficacy measures, and one was not a clinical study. Finally, we included 8 articles in this meta-analysis^17^,^18^,^19^,^20^,^21^,^22^,^23^,^24^ (Fig. 1).

Most of the analyzed studies (7/8) were randomized, double- or triple-blind and placebo-controlled. This meta-analysis comprised 665 patients with PAH receiving either statins (rosuvastatin, simvastatin, pravastatin or atorvastatin) (311 patients) or placebo (354 patients) for 6 months. In analyzed patients, PAH was idiopathic, heritable, associated with congenital heart disease, atrial-septal defects or connective tissue disease, and many of them suffered from chronic obstructive pulmonary disease. The PAH-related indices 6MWD, pulmonary arterial pressure, right atrial pressure, cardiac index and pulmonary vascular resistance were measured in 6, 6, 2, 4 and 2 analyzed studies, respectively (Table 1).

### Risk of bias assessment

The quality of the included studies assessed by the Newcastle-Ottawa Scale (NOS)^12^ is shown in Table 2.

### Effect of statin therapy on PAH-related indices

**Figure 2:**
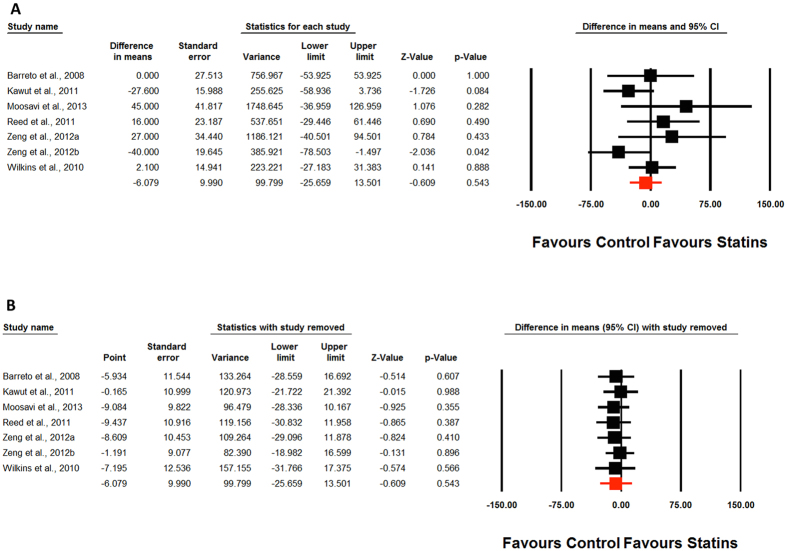
Forest plot detailing weighted mean difference and 95% confidence intervals for the impact of statin therapy on 6-min walking distance (Fig. 2A). Lower plot (Fig. 2B) shows leave-one-out sensitivity analysis.

**Figure 3:**
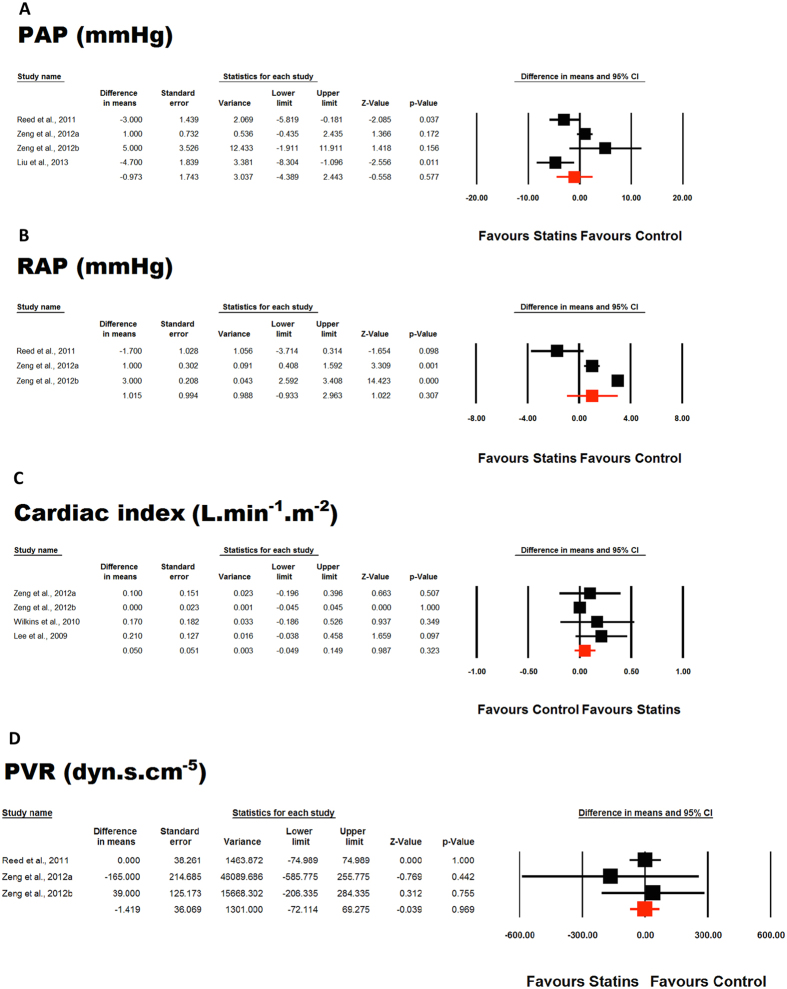
Forest plot detailing weighted mean difference and 95% confidence intervals for the impact of statin therapy on pulmonary arterial pressure (PAH) (Fig. 3A), right atrial pressure (RAP) (Fig. 3B), cardiac index (Fig. 3C), and pulmonary vascular resistance (PVR) (Fig. 3D).

Meta-analysis of included studies did not suggest any significant improvement in 6MWD by statin therapy (WMD: −6.08 m, 95% CI: −25.66, 13.50, *p* = 0.543; Q = 8.41, I^2^ = 28.64%) (Fig. 2A). This effect was robust in sensitivity analysis and the statistical significance was not influenced by any single study included in the meta-analysis (Fig. 2B). Likewise, none of the other indices including pulmonary arterial pressure (WMD: −0.97 mmHg, 95% CI: −4.39, 2.44, *p* = 0.577; Q = 14.64, I^2^ = 79.51%) (Fig. 3A), right atrial pressure (WMD: 1.01 mmHg, 95% CI: −0.93, 2.96, *p* = 0.307; Q = 44.88, I^2^ = 95.54%) (Fig. 3B), cardiac index (WMD: 0.05 L/min/m^2^, 95% CI: −0.05, 0.15, *p* = 0.323; Q = 3.82, I^2^ = 21.42%) (Fig. 3C), and pulmonary vascular resistance (WMD: −1.42 1.42 dyn*s/cm^5^, 95% CI: −72.11, 69.27, *p* = 0.969; Q = 0.69, I^2^ = 0%) (Fig. 3D) was significantly altered by statin therapy.

### Publication bias

**Figure 4:**
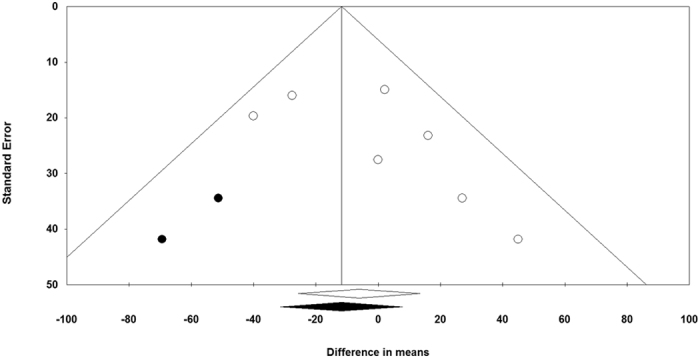
Funnel plot detailing publication bias in the studies reporting the impact of statin therapy on 6-min walking distance. Trim and fill method was used to impute for potentially missing studies. Open circles represent observed published studies; closed circles represent imputed unpublished studies.

The funnel plot of the study standard error by effect size (mean difference) for the meta-analysis of statin effects on 6MWD was asymmetric suggesting a potential publication bias (Fig. 4). Using “trim and fill” correction, two potentially missing studies were imputed on the left side of funnel plot, yielding an effect size of −11.72 m (95% CI: −31.27, 7.82). The results of Begg’s rank correlation (Kendall’s Tau with continuity correction = 0.38, *z* = 1.20, two-tailed *p*-value = 0.230) and Egger’s linear regression (intercept = 1.97, standard error = 1.25; 95% CI = −1.24, 5.18, *t* = 1.58, df = 5, two-tailed *p* = 0.176) tests excluded the possibility of publication bias in the meta-analyses.

## Discussion

To our knowledge, the current systematic review and meta-analysis is the first to evaluate the effects of statins on PAH. Contrary to the findings from some studies^18^,^25^, this meta-analysis of 8 studies did not suggest any significant improvement in 6MWD, pulmonary arterial pressure, right atrial pressure, cardiac index, and pulmonary vascular resistance by statin therapy. The lack of benefit was robust in sensitivity analysis and the statistical significance was not influenced by any single study included in the meta-analysis.

These findings are of clinical interest, since the use of statins in PAH treatment is thought to be beneficial due to their antiproliferative, anti-inflammatory, and pro-apoptotic pleiotropic effects as well as the ability to restore endothelial vasoactive mediator production^26^. Various studies linked statin effects with the pathogenesis of pulmonary hypertension and various biomarkers^27^, through analyzing the relation between statins, endothelin-1 and PAH^28^,^29^, statins, asymmetric dimethylarginine and PAH^30^,^31^,^32^, statins, D-dimers and PAH^33^,^34^, statins, von Willebrand factor antigen and PAH^35^,^36^ or fibrinogen and PAH^37^,^38^. Until now, the results obtained using statins on PAH parameters in different animal models have been contradictory. Some experimental studies suggested that statins might attenuate the development or even regress established experimental PAH by decreasing proliferation and increasing apoptosis of pathological smooth muscle cells in the medial walls and neointima of pulmonary arteries and reducing right ventricular hypertrophy^39^,^40^. Other experimental studies on murine models reported an inhibition of progression of emphysema and pulmonary hypertension related to tobacco exposure after statin therapy^41^,^42^. One experimental study showed that low doses of fluvastatin have beneficial effects on adventitial fibroblasts from chronic hypoxic animals through actions on the Rac1-p38 MAP kinase-signaling pathway^43^. Another experimental study showed that rosuvastatin improved ischemia-reperfusion injury by decreasing macrophage infiltration and up-regulating endothelial nitric oxide synthase^44^. In contrast, atorvastatin neither improved survival nor reduced PAH, vascular remodeling and right ventricular hypertrophy in an experimental study on a rat monocrotaline PAH model^45^. However, some experimental studies reported a decrease of right ventricular (RV) hypertrophy associated with statin therapy in PAH models^46^, but no study showed a reduction in pulmonary arterial pressure^47^,^48^. An experimental study on guinea pigs exposed to cigarette smoking during 6 months showed no effects of simvastatin on small airway remodeling^49^.

The data on humans from short-term randomized trials are few and contradictory. In an open-label observational trial, simvastatin was successfully used as adjunctive therapy in patients with PAH associated with various pathologies^25^. In another study, atorvastatin reduced pulmonary artery pressure and raised the migration and adhesion of endothelial progenitor cells (EPCs) in patients with chronic pulmonary heart disease^20^. Other studies described a decreased frequency of exacerbations and intubations^50^, a reduced decline of pulmonary function^51^ and decreased pulmonary pressures associated with improved exercise capacity after statin treatment^19^ in patients with chronic obstructive pulmonary diseases. A multicenter propensity score study on 2,363 patients showed for the first time that statins improve survival in PAH patients by decreasing one-year all-cause mortality^52^.

Similar to our results obtained in this meta-analysis, and in contrast to previous arguments, another study did not find any effect of statins on 6MWD^23^, but the short duration of this trial and background therapy of PAH patients may be potential causes for the lack of effects. Another trial on 220 PAH patients showed no benefit of atorvastatin on cardio-pulmonary hemodynamics, 6 MWD and survival at 6 months of treatment^24^, suggesting that atorvastatin should not be prescribed as specific treatment in PAH.

### Limitations

This meta-analysis has several limitations. *First,* the studies were relatively small and heterogeneous concerning study design, patients characteristics, major outcomes, PAH etiology and severity. *Second,* the patients included were classified in various functional classification groups. *Third,* the positive effects of statins in one study might have been overlapped by lack of effects of statins in the rest of studies since the effects of four statins (rosuvastatin, simvastatin, pravastatin and atorvastatin) were analyzed. *Fourth*, the patients received supportive drugs such as diuretics, warfarin, and digoxin, which could have influenced the results. The follow-up of the study was relatively short (6 months), so the long-term effects cannot be concluded and it is possible that longer therapy with statin might have been effective. Taking into account the given small sample size and short duration of the included studies, the meta-analysis could not also look at CVD outcomes that would be of the greatest interest. Finally, in the most of included studies PAH was diagnosed mainly based on echocardiography, which is also an important limitation.

*In conclusion*, the results of this meta-analysis of available studies did not show a statistically significant effect of statin therapy in the improvement of 6 MWD, pulmonary arterial pressure, right atrial pressure, cardiac index and pulmonary vascular resistance. Additional large, long term and well-designed trials are necessary to investigate the impact of statins for the treatment of PAH including their use as pulmonary vascular antiproliferative agents.

## Additional Information

**How to cite this article**: Rysz-Górzynska, M. *et al*. Efficacy of Statin Therapy in Pulmonary Arterial Hypertension: A Systematic Review and Meta-Analysis. *Sci. Rep.* **6**, 30060; doi: 10.1038/srep30060 (2016).

## Figures and Tables

**Table 1:** Main characteristics of the included studies.

First author’s name	Barreto *et al.*^17^	Kawut *et al.*^18^	Lee *et al.*^19^	Liu *et al.*^20^	Moosavi *et al.*^21^	Reed *et al.*^22^	Wilkins *et al.*^23^	Zeng *et al.*^24^
Year of publication	2008	2011	2009	2013	2013	2011	2010	2012
Study location	Brazil	USA	Taiwan	China	Iran	USA	Germany	China
Study design	Randomized, double-blind, placebo-controlled study	4-center, randomized, double-blind, placebo-controlled study	Randomized, double-blind parallel, placebo-controlled trial	Randomized, placebo-controlled trial	Randomized, triple-blind, parallel-group trial	Cohort, retrospective, cross-sectional, non-randomized	Randomized, double-blind, placebo-controlled trial	Randomized, double-blind and placebo-controlled trial
Inclusion criteria	Idiopathic or congenital heart disease-associated PAH (WHO functional class I–III) in the absence or presence of hypoxemia with a mean PAP >30 mmHg at rest.	Pts. >18 years of age with PAH (WHO functional class I–III).	COPD patients at age between 40 and 80 years with PAH, provided conditions were stable for at least 3 months.	60 to 85 years of age; in the stable chronic pulmonary heart disease with FEV1 ≤65% of the predicted value, the ratio of FEV1 to the forced vital capacity was ≤70% and PAH was >30 mmHg; and NYHA class I or II.	PAP >40 mmHg, able to doing 6-MWD test, obstructive pattern in PFT and functional NYHA class II or III.	COPD patients evaluated for lung transplantation with PAH with mean PAP at rest >25 mmHg.	Stable patients with class II or III PAH with mean PAP at rest >25 mm Hg, pulmonary capillary wedge pressure < 15 mmHg and pulmonary vascular resistance >3 Wood units; with a 6-MWD between 150–500 m at entry.	18–65 years of age pts. with Class 1 PAH defined as mean PAP >25 mmHg, pulmonary capillary wedge pressure < 15 mmHg and pulmonary vascular resistance >3 Wood units.
Number of participants in the statin group	30	32	27	33	24	34	19	112
Number of participants in the placebo group	30	33	26	35	21	78	23	108
Age of statin group	34.6 ± 12.3	51.7 ± 13.0	71 ± 8	66.2 ± 7.4	65 ± 11	58 ± 6	43.2 (19–67)	35 ± 13
Age of placebo group	33.7 ± 11.1	50.0 ± 14.3	72 ± 6	64.9 ± 8.2	68 ± 14	55 ± 9	49.1 (24–73)	37 ± 13
Gender of statin group	36 females, 24 males	26 females, 6 males	7 females, 20 males	13 females, 20 males	9 females, 15 males	17 females, 17 males	17 females, 2 males	79 females, 33 males
Gender of placebo group		30 females, 3 males	7 females, 19 males	12 females, 23 males	8 females, 13 males	49 females, 29 males	15 females, 8 males	65 females, 43 males
Body mass index (BMI) of statin group	NA	28.1 ± 7.7	22 ± 2	NA	NA	25 ± 5	24.4 ± 4.7	NA
Body mass index (BMI) of placebo group	NA	28.6 ± 7.8	23 ± 1	NA	NA	24 ± 5	26.3 ± 3.3	NA
Type of statin therapy	rosuvastatin	simvastatin	pravastatin	atorvastatin	atorvastatin	atorvastatin, simvastatin	simvastatin	atorvastatin
Duration of statin therapy	6 months	6 months	6 months	6 months	6 months	NA	24 weeks	24 weeks
Baseline 6MWD (m) values in statin group	416 ± 103	422.9 ± 101.4	NA	NA	238 ± 124	259 ± 94	381 ± 69 (198–498)	355 ± 74
Follow-up 6MWD (m) values in statin group	no increase	no difference	NA	NA	339 ± 155	NA	increased by 3.1 ± 34.5	decreased by 52 (−103– −1)** increased by 10 (−19–39)***
Baseline 6MWD (m) values in placebo group	415 ± 110	418.3 ± 131.7	NA	NA	284 ± 100	243 ± 120	386 ± 110 (120–600)	346 ± 84
Follow-up 6MWD (m) values in placebo group	no increase	NA	NA	NA	340 ± 106	NA	increased by 1.0 ± 57.0	decreased by 79 (−126– −33)** increased by 50 (31–69)***
Baseline values of pulmonary arterial pressure (mmHg) in statin group	53 ± 16	NA	47 ± 8	52.7 ± 8.1	NA	26 ± 7	55.8 ± 10.3 (42–71)	69 ± 19
Baseline values of pulmonary arterial pressure (mmHg) in placebo group		NA	47 ± 7	51.7 ± 7.9	NA	29 ± 7	55.7 ± 12.5 (30–81)	66 ± 20
Follow-up values of pulmonary arterial pressure (mmHg) in statin group	NA	NA	40 ± 6	45.4 ± 6.8	NA	decreased by 5.2 mmHg	NA	increased by 4 (0–7)* increased by 11 (4–19)** not changed 0 (−5–4)***
Follow-up values of pulmonary arterial pressure (mmHg) in placebo group	NA	NA	46 ± 7	49.1 ± 7.3	NA	NA	NA	increased by 3 (−1–6)* increased by 10 (4–16)** decreased by 5 (−10–0)***
Baseline values of right atrial pressure in statin group	NA	NA	NA	NA	NA	8.6 ± 5	NA	9 ± 6
Baseline values of right atrial pressure in placebo group	NA	NA	NA	NA	NA	10.3 ± 5	NA	10 ± 6
Follow-up values of right atrial pressure in statin group	NA	NA	NA	NA	NA	NA	NA	increased by 3 (2–5)* increased by 6 (2–9)** increased by 3 (1–5)***
Follow-up values of right atrial pressure in placebo group	NA	NA	NA	NA	NA	NA	NA	increased by 3 (2–5)* increased by 5 (3–7)** increased by 0 (−2–2)***
Baseline values of cardiac index (l/m^2^) in statin group	NA	NA	4.09 ± 0.49	NA	NA	2.8 ± 0.6	2.6 ± 0.65	2.4 ± 0.8
Baseline values of cardiac index (l/m^2^) in placebo group	NA	NA	4.33 ± 0.55	NA	NA	2.9 ± 0.7	2.5 ± 0.7	2.6 ± 1
Follow-up values of cardiac index (l/m^2^) in statin group	NA	NA	4.23 ± 0.33	NA	NA	NA	increased by 0.12 ± 0.58	decreased by 0.1 (–0.2–0)* decreased by 0.3 (−0.5–0)** not changed 0 (−0.1–0.2)***
Follow-up values of cardiac index (l/m^2^) in placebo group	NA	NA	4.26 ± 0.38	NA	NA	NA	decreased by 0.05 ± 0.59	decreased by 0.2 (−0.3–0)* decreased by 0.4 (−0.6– −0.2)** not changed 0 (−0.3–0.3)***
Baseline values of pulmonary vascular resistance (dyn*s*cm^−5^) in statin group	NA	NA	NA	NA	NA	5.0 ± 2.6	NA	1633 ± 745
Baseline values of pulmonary vascular resistance (dyn*s*cm^−5^) in placebo group	NA	NA	NA	NA	NA	5.0 ± 2.2	NA	1456 ± 699
Follow-up values of pulmonary vascular resistance (dyn*s*cm^−5^) in statin group	NA	NA	NA	NA	NA	NA	NA	increased by 203 (42–364)* increased by 440 (119–762)** increased by 46 (−151–243)***
Follow-up values of pulmonary vascular resistance (dyn*s*cm^−5^) in placebo group	NA	NA	NA	NA	NA	NA	NA	increased by 287 (132–442)* increased by 605 (313–897)** increased by 8 (−150–165)***

Data are presented as mean ± SD and range in brackets.

ABBREVIATIONS: 6-MWD – 6 minutes walk distance; COPD – chronic obstructive pulmonary disease; FEV1 – forced expiratory volume in one second % of vital capacity; NYHA - New York Heart Association; PAH – pulmonary arterial hypertension; PAP – pulmonary arterial pressure; PFT - pulmonary function test; WHO – world health organization;

*mean change; **change in patients with idiopathic pulmonary arterial hypertension and pulmonary arterial hypertension associated with connective tissue disorder; ***change in patients with pulmonary arterial hypertension associated with congenital heart disease.

**Table 2:** The quality of the studies assessed using Newcastle-Ottawa Scale.

Domain and Topic (max. 10 stars)									
Author	Year	Selection (max. 5 stars)				Comparability (max. 2 stars)	Outcome (max. 3 stars)		Total
		Representativeness of the sample	Sample size	Non-responders	Ascertainment of the exposure	Subjects in different outcome groups are comparable	Assessment of Outcome	Choice of statistical tests	
**Barreto** ***et al.***^17^	2008	★	★		★	★	★★	★	7
**Kawut** ***et al.***^18^	2011	★	★	★	★★	★	★★	★	9
**Lee** ***et al.***^19^	2009	★	★	★	★★	★	★★	★	9
**Liu** ***et al.***^20^	2013	★	★		★★	★	★	★	7
**Moosavi** ***et al.***^21^	2013	★		★	★★	★	★★	★	8
**Reed** ***et al.***^22^	2011	★	★		★★	★	★★	★	8
**Wilkins** ***et al.***^23^	2010	★		★	★★	★	★★	★	8
**Zeng** ***et al.***^24^	2012	★	★	★	★★	★	★★	★	9
